# Assessment of Immune Cell Populations in Tumor Tissue and Peripheral Blood Samples from Head and Neck Squamous Cell Carcinoma Patients

**DOI:** 10.1155/2021/2328218

**Published:** 2021-10-15

**Authors:** Ana Caruntu, Liliana Moraru, Mihaela Surcel, Adriana Munteanu, Cristiana Tanase, Carolina Constantin, Sabina Zurac, Constantin Caruntu, Monica Neagu

**Affiliations:** ^1^Department of Oral and Maxillofacial Surgery, “Carol Davila” Central Military Emergency Hospital, 010825 Bucharest, Romania; ^2^Department of Oral and Maxillofacial Surgery, Faculty of Dental Medicine, “Titu Maiorescu” University, 031593 Bucharest, Romania; ^3^Immunology Department, Victor Babes National Institute of Pathology, 050096 Bucharest, Romania; ^4^Faculty of Biology, University of Bucharest, Bucharest 76201, Romania; ^5^Biochemistry Laboratory, Victor Babes National Institute of Pathology, 050096 Bucharest, Romania; ^6^Faculty of Medicine, “Titu Maiorescu” University, 031593 Bucharest, Romania; ^7^Department of Pathology, Colentina University Hospital, Bucharest 020125, Romania; ^8^Department of Pathology, “Carol Davila” University of Medicine and Pharmacy, Bucharest 020125, Romania; ^9^Department of Physiology, Carol Davila University of Medicine and Pharmacy, 050474 Bucharest, Romania; ^10^Department of Dermatology, Prof. N.C. Paulescu National Institute of Diabetes, Nutrition and Metabolic Diseases, 011233 Bucharest, Romania

## Abstract

Head and neck squamous cell carcinoma (HNSCC) is a common type of cancer worldwide. Strong connections have been revealed between immune cells and the pathogenesis of HNSCC. Important differences regarding the levels of immune cell subpopulations in both peripheral circulation and tumor microenvironment were emphasized, with some of them having prognostic significance. In our study, we performed an analysis of immune changes in the tumor tissue and the peripheral blood of untreated HNSCC patients, investigating the proportions of different immune cell populations in these two compartments. The local infiltrating lymphocytes were mainly cytotoxic T cells (CD8^+^). We have also revealed an increased level of B lymphocytes (CD19^+^) in the tumor microenvironment. In peripheral blood, the most important lymphocyte subtype was represented by the helper T lymphocytes (CD4^+^). We also found an increased proportion of circulating NK cells (CD56^+^). Our results showed significant differences between all investigated lymphocyte subtypes in the peripheral blood and the tumor tissue of untreated HNSCC patients, suggesting that the local and systemic expressions of antitumor immune responses are different and that investigation of immune cell proportions in peripheral circulation has different cues that do not reflect the immune infiltrate pattern within the tumor microenvironment. Further studies are necessary to unveil the complex interplay involving local and systemic events in the immune system's fight against cancer.

## 1. Introduction

Head and neck squamous cell carcinoma (HNSCC) is an epithelial type of cancer, with a high prevalence and an increasing incidence worldwide. The immune inflammatory factors are among the most important actors in the onset and progression of cancer [1–6], and numerous studies support important connections between immune cells, especially lymphocytes, and the pathogenic mechanisms of HNSCC [7–11].

Progression from early stages to advanced locoregional disease is associated with a significant alteration in the number and function of immune cell populations in peripheral blood, correlated with the inability of the immune system to limit the evolution of the tumor, facilitating tumor growth [7]. Moreover, tumor-infiltrating immune cells have attracted a special attention in scientific research, due to their impact on tumor development and progression [12, 13]. Multiple research findings suggest that there is a close relationship between local tumor inflammatory infiltrate, local disease control, and patient survival [7, 10]. However, the complexity of the immune carcinogenic interplay in HNSCC is not fully unveiled yet. Various populations and subpopulations of lymphocytes, such as cytotoxic T lymphocytes (CD8^+^), helper T lymphocytes (CD4^+^), and B lymphocytes, along with other types of immune cells, such as NK cells, acting in the tumor microenvironment may exert coordinated or sometimes even contrary responses [7, 10].

Peritumoral infiltration rich in total T lymphocytes (CD3^+^), as well as particularly in cytotoxic T lymphocytes (CD8^+^), main actors in tumor surveillance, was correlated with a favorable prognosis in HNSCC [14]. Helper T lymphocytes (CD4^+^) mediate antitumor immunity [15]; however, in HNSCC, the prognostic significance of their presence in the tumor microenvironment is not yet settled [16].

The role of infiltrating B lymphocytes in HNSCC is still uncertain. However, there are results showing a better prognosis associated with an increased density of intratumoral B cells together with a high infiltrate of cytotoxic T lymphocytes (CD8^+^) [17] supporting further studies in this direction.

Natural killer (NK) CD56^+^ cells are leading actors of the innate immune system, having an effective role in tumor immunosurveillance, alongside their equivalents in the adaptive immune system—the cytotoxic T cells (CD8^+^) [7, 18, 19]. Several studies have emphasized an improved disease control and a better outcome associated with an increased intratumor density of NK cells in HNSCC patients [20, 21]. However, other research has revealed tumor resistance strategies, suggesting a supporting role of NK cells in tumor progression [22, 23].

In HNSCC, a high variability of immune cell subpopulations was observed, partially correlating with the prognosis of patients. The information presented above demonstrates that a real representation of the antitumor response capacity is a topic of major interest. Moreover, an important issue is whether in HNSCC the proportion of circulating immune cells provides a relevant picture of the immune infiltrate in the tumor microenvironment or each of these two immune-related investigations portrays different points of view of a complex process with distinct local and systemic expressions.

In our study, we have investigated the differences between the distribution of immune cell populations in tumor tissue and peripheral blood samples from treatment-naïve HNSCC patients.

## 2. Materials and Methods

### 2.1. Study Protocol

In this study, we have included patients with operable forms of HNSCC treated in the Department of Oral and Maxillofacial Surgery, “Carol Davila” Central Military Emergency Hospital, Bucharest. The study was conducted in accordance with the Declaration of Helsinki (1964), with the approval of the Local Ethics Committee (No. 25/November 27, 2017). All patients included in the study were informed of the study protocol and signed the informed consent form.

All patients met the following inclusion criteria: histopathological confirmed diagnosis of HNSCC, in operable stages, that did not receive any previous treatment. Patients with unresectable or metastatic tumors, with other types of malignancy, immunological conditions, and other severe, decompensated conditions or with incomplete medical records were excluded.

All patients underwent a thorough preoperative evaluation, which included, in addition to the usual investigations, the collection of peripheral blood samples to determine circulating lymphocyte subtypes using the flow cytometry technique.

After radical resection of the tumor, histopathological examination of the excision specimen was performed, with a subsequent immunohistochemical study.

Surgical treatment was followed by oncological therapy and/or active follow-up program, according to national therapeutic guidelines.

### 2.2. Flow Cytometry Analysis

Based on the expression of surface markers, immunophenotyping allows quantification by flow cytometry of main lymphocyte subsets from whole hemolyzed blood: T lymphocytes (CD45+CD3+), B lymphocytes (CD45+CD3-CD19+), helper T lymphocytes (CD45+CD3+CD4+), suppressor/cytotoxic T lymphocytes (CD45+CD3+CD8+), and NK cells (CD45+CD3-CD16+CD56+).

In order to determine the percentages of these subsets, a BD Multitest IMK Kit (IVD) (Becton Dickinson) was used. EDTA-anticoagulated whole peripheral blood was incubated with a mixture of monoclonal antibodies (CD3-FITC/CD8-PE/CD45-PerCP/CD4-APC; CD3-FITC/CD16+CD56-PE/CD45-PerCP/CD19-APC) for 15 min at room temperature and in the dark, followed by red blood cell lysis and flow cytometry analysis (BD FACSCanto II, Becton Dickinson). BD FACSCanto clinical software was used for sample acquisition and data analysis; daily check-up of cytometer performances was performed using 7-Color Setup Beads (BD Biosciences).

### 2.3. Histopathologic Examination

The surgical specimens were immediately immersed in 10% buffered formalin and sent for histopathologic diagnosis. The macroscopic examination and selection of the fragments were performed according to the national and international protocols; further, the tissue fragments were manually processed and paraffin embedded. The paraffin blocks were cut with a semiautomated Rotary Microtome Leica RM2245; 3 *μ* sections were obtained, taken on regular slides for routine and special stains and on precoated slides for immunohistochemical tests.

Several immunohistochemical (IHC) stains were performed for CD4, CD8, CD19, and CD56 (see Table 1). Novolink Polymer (Leica/Novocastra) and DAB chromogen were used as the detection system.

Immunohistochemical analysis was performed using an Olympus BX41 microscope; CD4, CD8, CD19, and CD56 were evaluated in lymphoid cells. All the markers were evaluated as the number of positive cells per high-power field (HPF) (0.55 mm in diameter) both in the intratumor location and in the invasion front, with the number of the positive cells being appreciated in hot spot by counting 10 adjacent HPF.

### 2.4. Statistical Analysis

For statistical analysis, we used GraphPad Prism 9 (GraphPad Software, Inc., San Diego, CA, USA). We evaluated the normality of the data distribution using the Kolmogorov-Smirnov test. The differences between the lymphocyte subtypes in peripheral blood and tumor tissue were assessed by the paired *t*-test (normal distribution) or the Wilcoxon test (nonnormal distribution). The results are presented as mean ± standard deviation (SD); *p* values < 0.05 were considered statistically significant.

## 3. Results

### 3.1. Patient Characteristics

A total of 10 patients with operable HNSCC were included in the study, 7 males and 3 females, with a mean age of 67.6 ± 14.25 years, ranging from 45 to 86 years old. The primary tumor was staged from T1 to T4a and involved different head and neck subsites: lower lip, buccal mucosa, tongue, gingiva, and retromolar mucosa. In three patients, cervical lymph node involvement was confirmed. Histology analysis of the specimen revealed that 50% of the lesions were moderately differentiated, 40% were well differentiated, and 10% were poorly differentiated. Descriptive data of the patient cohort is presented in Table 2.

### 3.2. Immunohistochemical Analysis of Resected HNSCC Tumors

**Figure 1:**
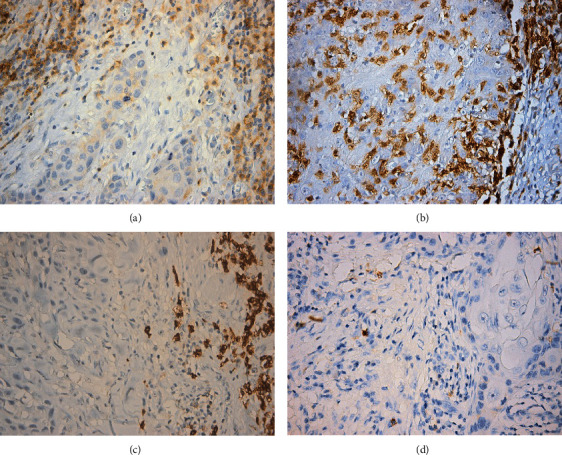
Immune cell subtypes evaluated within the inflammatory infiltrate in the tumor microenvironment of head and neck squamous cell carcinoma (HNSCC). (a) (CD4^+^) T lymphocytes within the peritumoral inflammatory infiltrate. (b) Numerous (CD8^+^) T cells within the intratumor inflammatory infiltrate (E). (c) (CD19^+^) B lymphocytes, mostly forming small aggregates present within the peritumoral inflammatory infiltrate. (d) Few (CD56^+^) NK cells within the peritumoral inflammatory infiltrate. Original magnification ×400.

Immune cell subtypes evaluated within the inflammatory infiltrate in the tumor microenvironment focused on T cell subpopulations (CD4^+^, CD8^+^), total B cells (CD19^+^), and NK cells (CD56^+^) (Figure 1 and Table 3). The number of positive cells in the tumor microenvironment was evaluated for all the tumor samples. We traced different distribution patterns of lymphocytes within the tumor tissue. Thus, T lymphocytes (CD4^+^ and CD8^+^) represented the main immune cell type in the peritumoral infiltrate, with cytotoxic T lymphocytes (CD8^+^) being the vast majority of these cells, with a rigorously spread pattern within the tumor tissue. The B cell population (CD19^+^) represented almost 40% of the tumor immune infiltrate analyzed in our study, following a distinct pattern of organization, in small peritumoral aggregates. NK cells (CD56^+^) were present as isolated cells, rarely found in the tumor microenvironment.

### 3.3. Comparative Investigation of Immune Cell Populations in Tumor Tissue and Peripheral Blood Samples of HNSCC Patients

**Figure 2:**
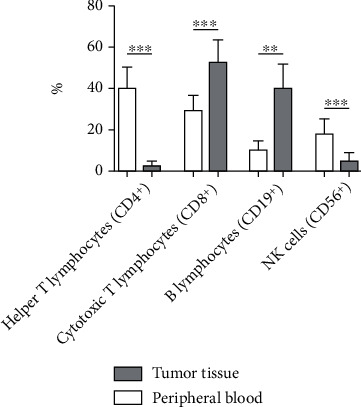
Percentual representation of immune cell populations in tumor tissue and peripheral blood samples of HNSCC patients. The error bars represent the standard deviation; ^∗∗^*p* < 0.01; ^∗∗∗^*p* < 0.001.

Analysis of lymphocyte subtypes showed significant differences between peripheral blood and tumor tissue in HNSCC patients (see Table 3 and Figure 2).

In peripheral blood, the most important lymphocyte subtype was represented by helper T lymphocytes (CD4^+^), with a proportion significantly higher than the tumor tissue (*p* < 0.0001).

In contrast, cytotoxic T lymphocytes (CD8^+^) represented over half of the lymphocyte populations in the tumor infiltrate and were markedly increased compared to peripheral circulation (*p* = 0.0002).

Also, the level of B lymphocytes (CD19^+^) was significantly higher in tumor tissue than in peripheral blood (*p* = 0.0020).

As for NK cells (CD56^+^), their proportion was much lower in the tumor infiltrate compared to peripheral circulation (*p* = 0.0003).

## 4. Discussion

Alteration of cellular and humoral immune responses has been indicated as important players in the development and progression of HNSCC [7, 24]. Systemic immunity may affect the clinical evolution of head and neck cancer [25], and the local immunity is essential in the control of tumor growth and invasion capacity [7, 24].

In our study, we performed an analysis of immune changes in the peripheral blood and the tumor tissue of untreated HNSCC patients, investigating the distribution of different immune cell populations in these two compartments.

The local infiltrating lymphocytes were mainly cytotoxic T cells (CD8^+^). We have also revealed an increased level of B lymphocytes (CD19^+^) in the tumor microenvironment.

CD8^+^ lymphocytes are main effectors in antitumor protection [26], and other studies have also revealed their higher levels in the inflammatory infiltrate of HNSCC [27, 28]. An increased number of CD8^+^ cytotoxic lymphocytes in the tumor microenvironment also carry a favorable prognostic significance in HNSCC [10, 14, 29, 30].

Concerning the role of the tumor infiltrating B lymphocytes, opinions are still divided, highlighting both beneficial and unfavorable aspects regarding their antitumor immune effects and their prognostic impact [7, 17, 31]. An interesting finding is the close interconnection between B lymphocytes (CD19^+^) and cytotoxic T cells (CD8^+^) in the tumor microenvironment with a favorable prognostic impact in HNSCC [31]. The role of B cells within the tumor is complex. In other types of cancer, it was established that B cells promote tumor cell clearance through the release of specific immunoglobulins that enhance T cell-mediated response. Nevertheless, B cells through their subpopulation (Bregs) can also suppress antitumor immune response by immunosuppressive cytokines which regulate T cells, NK cells, and myeloid-derived suppressor cells (MDSC). They also can secrete pathological antibodies or promote angiogenesis [32]. Our findings regarding the high percentage of B cells within the tumor site and their clustering suggest that in our tumor samples, there is an active immune response ongoing between the infiltrating immune cells. The lower percentage of circulating B cells accounts for their drainage toward the tumor tissue as part of the local immune response.

In our study, helper T lymphocytes (CD4^+^) were the most important type of immune cells found in peripheral circulation. Helper T lymphocytes are leading actors in the initiation and modulation of the antitumor immune responses and are the main immune cell subtype present in peripheral blood [15, 33, 34]. However, a study comparing lymphocyte subtypes in HNSCC with healthy control subjects reported a decreased level of circulating helper T cells (CD4^+^) in the test group [35]. Our results indicate a clear disproportion of CD4^+^ T lymphocytes between circulation and tumor tissue that suggests several issues related to HNSCC: a reduced intratumoral cooperation between CD4^+^ and CD8^+^ cells, which has sustained tumor proliferation, and a decreased migration of CD4^+^ lymphocytes towards the tumor site due to still unknown extracellular and/or intrinsic factors.

A high proportion of NK cells (CD56^+^) was identified in the peripheral circulation of HNSCC patients. As NK cells (CD56^+^) are effectors of the innate immune system with potent antitumor functions, finding a high percentage in the patient's circulation is a good indicator for HNSCC patients. Moreover, another study has indicated an increase in NK cell level after the treatment of HNSCC [36]. It is worth mentioning that alteration of their cytotoxic functions were associated with oral tumors, and inhibitory actions of NK cells were also emphasized in both peripheral circulation and tumor microenvironment in HNSCC [37, 38].

Our results showed significant differences between all investigated lymphocyte subtypes in the peripheral blood and the tumor tissue, bringing new evidence in a vast area of research, with complex mechanisms still not clearly understood [1–4, 7, 8]. In the scientific literature, there is little information about the differences between the distributions of immune cell populations in tumor tissue and peripheral blood from HNSCC patients, and most of them refer only to T lymphocytes.

An interesting study evaluating the subgroups of T lymphocytes and the immune regulatory mechanisms in untreated HNSCC patients [39] showed that T lymphocytes represent the vast majority of infiltrating lymphocytes in the tumor, with their proportion being higher in the tumor tissue than in the peripheral blood in HNSCC patients. The level of cytotoxic T lymphocytes (CD8^+^) tended to be higher in the tumor than in the peripheral circulation; however, the difference was not statistically significant. In contrast, the percentage of CD4^+^ T lymphocytes was significantly higher in peripheral blood than in tumor tissue. In addition, the study showed that the tumor microenvironment of HNSCC has a strong infiltration of T lymphocytes with an effector memory phenotype, with the regulatory T cells being significantly increased [39].

Other research has indicated different aspects than those revealed by our results. A correlation analysis of peripheral blood lymphocytes and tumor infiltrating lymphocytes in patients with oral squamous cell carcinoma found significant correlations regarding the level of total T lymphocytes (CD3^+^), helper T lymphocytes (CD4^+^), and cytotoxic T lymphocytes (CD8^+^), suggesting that circulating T cell levels could be an indicator for the local T cell-mediated antitumor responses [38].

A comparative assessment of lymphocyte subtypes in tumor tissues, lymph nodes, and peripheral blood of patients with HNSCC revealed significant differences regarding the local, regional, and systemic immune responses [24]. Thus, the level of CD4^+^ T lymphocytes was higher in the peripheral circulation than in the tumor tissue, a result similar to our research. However, regarding the level of CD8^+^ T lymphocytes, although an increasing trend was observed in the tumor compared to peripheral blood, the differences were not statistically significant. In addition, the same study revealed an altered cytotoxic activity of lymphocytes in the tumor microenvironment and a decreasing tendency of NK cells (CD56^+^) in the tumor tissue compared to peripheral circulation, but again without reaching the threshold of statistical significance [24]. Acknowledging the limitations of our research regarding the low number of investigated patients, the differences from other studies could be explained by the different locations of the tumors, selection of patients in different stages of the disease, and inclusion in other studies of patients previously exposed to oncological treatment with radiotherapy and/or chemotherapy.

## 5. Conclusions

Our study revealed significant differences between the levels of lymphocyte subtypes in peripheral circulation and the tumor tissue of untreated HNSCC patients, suggesting that the local and systemic expressions of antitumor immune responses are different and that investigation of immune cell proportions in peripheral circulation has different cues that do not reflect the immune infiltrate pattern within the tumor microenvironment. Further studies are necessary to unveil the complex interplay involving local and systemic events in the immune system's fight against cancer.

## Figures and Tables

**Table 1:** Specific details of immunohistochemical markers.

Marker	Clone	Host	Dilution	Pretreatment^∗^	Producer
CD3	LN10	Mouse	1 : 500	HIER, citrate, pH 6	Leica
CD19	BT51E	Mouse	1 : 100	HIER, citrate, pH 6	Leica
CD4	4B12	Mouse	1 : 200	HIER, EDTA, pH 8	Leica
CD8	4B11	Mouse	1 : 100	HIER, EDTA, pH 8	Leica
CD56	CD564	Mouse	1 : 200	HIER, citrate, pH 6	Leica

^∗^HIER: heat-induced epitope retrieval.

**Table 2:** Clinical and histopathological details of HNSCC patients.

Patient #	Gender	Age	Tumor location	Clinical staging	Histological differentiation
1	M	80	Buccal mucosa	T2N0M0	WD
2	M	60	Retromolar mucosa	T3N2M0	PD
3	M	80	Lower lip	T1N0M0	WD
4	M	51	Tongue	T2N2M0	WD
5	M	65	Gingiva	T2N0M0	MD
6	F	85	Lower lip	T2N0M0	MD
7	F	86	Lower lip	T1N0M0	WD
8	F	54	Tongue	T2N0M0	MD
9	M	45	Buccal mucosa	T3N1M0	MD
10	M	71	Gingiva	T4aN1M0	MD

M: male; F: female; WD: well differentiated; MD: moderately differentiated; PD: poorly differentiated.

**Table 3:** Proportion of lymphocyte subtypes in tumor tissue and peripheral blood samples of HNSCC patients.

Variable (%)	Peripheral blood		Tumor tissue		*p* value
	Mean	SD	Mean	SD	
Helper T lymphocytes (CD4^+^)	40.10	10.19	2.35	2.5	<0.0001^#^
Cytotoxic T lymphocytes (CD8^+^)	29.10	7.39	52.72	10.87	0.0002^#^
B lymphocytes (CD19^+^)	9.60	4.93	39.77	11.80	0.0020^∗^
NK cells (CD56^+^)	18.10	6.62	5.16	3.45	0.0003^#^

^#^Paired *t*-test; ^∗^Wilcoxon test.

## Data Availability

The datasets used and/or analyzed during the present study are available from the corresponding author.
